# Self-Medication Among Pregnant Women: Prevalence and Associated Factors

**DOI:** 10.3389/fphar.2021.659503

**Published:** 2021-12-03

**Authors:** Gabriela Pereira, Fernanda Garanhani Surita, Amanda Canato Ferracini, Cinthia de Souza Madeira, Letícia Silva Oliveira, Priscila Gava Mazzola

**Affiliations:** ^1^ Faculty of Pharmaceutical Sciences, University of Campinas, Campinas, Brazil; ^2^ School of Medical Sciences, Department of Obstetrics and Gynecology, University of Campinas, Campinas, Brazil; ^3^ Graduate Program in Medical Sciences, School of Medical Sciences, University of Campinas, Campinas, Brazil; ^4^ Graduate Program in Gerontology, School of Medical Sciences, University of Campinas, Campinas, Brazil

**Keywords:** self-medication, pregnancy, women’s health, medication use, antenatal care

## Abstract

**Objectives:** The pregnancy period, with its peculiarities and specific symptoms that may or may not be physiological, can lead to medication use through prescription or even self-medication. This study aimed to assess self-medication practices among pregnant women, the most used medications, symptoms reported, and factors associated with this practice.

**Methods:** This was a cross-sectional study conducted with pregnant women with an antenatal care (ANC) appointment in a tertiary teaching hospital referral in women’s health. From April 2019 to February 2020, 297 pregnant women were interviewed. Self-medication was considered as the use of any medicine (including medicinal plants (MPs), herbal products, and vitamins) without a medical or dental prescription. The period considered to assess self-medication practice was the last 60 days prior to the study interview.

**Results:** Among the 297 women interviewed, 107 (36.0%) had practiced self-medication in the previous 60 days. Acetaminophen was the most used medication, and headache was the most frequent symptom reported by self-medicated pregnant women. Pregnant women with high-school (73 (68.2%) (OR = 2.52; 95% CI 1.17–5.43; p = 0.018)) or university-level (23 (21.5%) (OR = 2.82; 95% CI 1.15–6.94; p = 0.024)) education had a higher risk of practicing self-medication when compared to women with lower education. Women in the first gestational trimester (35 (32.7%) (OR = 3.61; 95% CI 1.64–7.96; p = 0.002)) and with two or more pregnancies (87 (81.2%) (OR = 1.96; 95% CI 1.07–3.60; p = 0.029)) were more likely to practice self-medication than pregnant women in the second or third gestational trimester and in the first pregnancy, respectively.

**Conclusion:** Self-medication was practiced by a considerable proportion of our sample, with the majority being OTC drugs. The factors associated with self-medication can help to improve prevention strategies regarding self-medication during pregnancy.

## Introduction

Self-medication is defined by the World Health Organization (WHO) as the “selection and use of medicines by individuals to treat self-recognized illness or symptoms,” including medicinal plants (MPs) and herbal products (WHO, 1998).

Pregnant women report a variety of symptoms throughout their pregnancy due to physiological and anatomical changes (Moya et al., 2014). Headache, nausea, vomiting, and edema are frequent symptoms during pregnancy and are commonly related to self-medication practice during this period (Bohio et al., 2016; Cabut et al., 2017; Botyar et al., 2018; Zewdie et al., 2018). Beyond the symptoms presented, self-medication during pregnancy is typically motivated by easy access to medicines, previous medication experience (Beyene and Beza, 2018), and time saving (Zewdie et al., 2018) and are associated with sociodemographic factors (Liao et al., 2015; Marwa et al., 2018; Zewdie et al., 2018), previous maternal illness (Abeje et al., 2015), and poor knowledge regarding medication use and its risks (Beyene and Beza, 2018).

Studies have shown substantial differences in the prevalence of self-medication during pregnancy (Beyene and Beza, 2018; Marwa et al., 2018; Zewdie et al., 2018; Atmadani et al., 2020). One study performed in the Netherlands found a self-medication prevalence of 12.5%, whereas research performed in Nigeria and Iran reported a prevalence of 63.8 and 30.6%, respectively (Yusuff and Omarusehe, 2011; Verstappen et al., 2013; Afshary et al., 2015). This difference could be related to the study setting, the concept of self-medication considered by authors, and the recording period used to assess self-medication practice.

According to the gestational age at the exposure time and the dose administrated, the use of a teratogenic agent during pregnancy can result in varying outcomes, such as fetal death, morphologic malformations, or physiological abnormalities (Cohlan, 1963; Shepard, 1979; Mazzu-Nascimento et al., 2017). Although exposure to teratogenic effects is responsible for a minor number of birth defects, it can be considered a public health issue once this child will require specialized attention throughout their life (Brazil, 2013; Mazzu-Nascimento et al., 2017).

There is a lack of literature regarding self-medication among pregnant women living in Brazil. This study will help to understand the current scenario in an urban city of Brazil and could assist future research in the field. The findings regarding the factors associated with self-medication during pregnancy will assist health professionals in preventing self-medication among pregnant women.

Considering the easy access to medicines and the symptoms associated with pregnancy changes, this study aims to evaluate the self-medication prevalence among pregnant women, the associated factors, most common medications used, symptoms reported, and motivational factors related to this practice among pregnant women in an urban city of Brazil.

## Methods

### Study Design and Setting

A cross-sectional study was conducted in a high-risk antenatal care (ANC) clinic at the Women’s Hospital of the University of Campinas, São Paulo, Brazil. This is a public tertiary teaching hospital specializing in women’s health; it has 142 beds and performs an average of 200 deliveries per month. Pregnant women can access the high-risk ANC service through a medical referral.

### Participants and Inclusion and Exclusion Criteria

From April 2019 to February 2020, 297 pregnant women were interviewed. The inclusion criteria were pregnant women in their first appointment at the ANC at the Women’s Hospital, aged 18 years or more, with no restriction regarding their gestational age. The study took place in a city that receives immigrants from different countries, and the exclusion criterion selected was as follows: “does not speak fluent Portuguese (local language).

### Data Collection Procedures

Pregnant women were selected by simple random sampling according to their availability in the waiting room. The data collection was performed in a private room, through face-to-face interviews, using semistructured questionnaires developed by the research team. Questionnaires were developed based on the literature (Adanikin and Awoleke, 2017; Beyene and Beza, 2018; Marwa et al., 2018) and they were analyzed by two professors with expertise in the research area through face, content, construct, and validity to assess the comprehensiveness, readability, and clarity of the instrument. A pretest was conducted with five pregnant women to assess the instruments’ validity and reliability. Changes regarding the organization of the instruments (e.g., the creation of a table to organize the results) were made to improve the questionnaires’ quality. The participants included in the pretest were not included in the final analysis. The participants answered two questionnaires: 1) sociodemographic, gestational, and life habits and 2) self-medication practice during pregnancy. All procedures were carried out according to the Helsinki Declaration. All Strengthening the Reporting of Observational Studies in Epidemiology (STROBE) statement items were followed and confirmed in this manuscript.

### Variables

#### Dependent Variables

The outcome measure was the practice of self-medication. Self-medication was considered as the use of any medicine, MP, phytotherapy, or vitamin used without a medical or dental prescription and the use of the medicine prescribed to another person. The period considered to assess self-medication during pregnancy was the previous 60 days prior to the first appointment at the Women’s Hospital, and this period was used to minimize recall bias.

For pregnant women who gave a positive answer to self-medication practice, the medications used during self-medication were assessed, asking “Which medications did you use during self-medication?” The medications informed were registered and separated into three groups (WHO, 1998): over-the-counter drugs (OTC) (Moya et al., 2014); prescription-only medicines (POM); and (Bohio et al., 2016) MPs, phytotherapy, and vitamins. MPs were considered as the administration of any plant or part of it without the use of any specific fabrication process (e.g., chamomile tea). Phytotherapy was defined as the use of a plant or part of it through a specific formulation (e.g., passionflower capsule). Vitamins considered in self-medication were both combined or isolated vitamins. The indication for self-medication practice was assessed by asking the question, “Which symptom did you feel to practice self-medication?”

Motivational factors were assessed by asking pregnant women: “Why did you practice self-medication?” Different options were given (considered a simple health problem, practicality, and lack of health-system access). There was an option—“Other”—to classify the answers that were not contemplated in the above list. The source or person who indicated a self-medication practice was assessed by the question “From whom was the indication for self-medication obtained?” Different options were given (own account, neighbor or friend, leftover from a previous treatment, family member, pharmacy, or internet). Another option—“Other”—was available to classify those answers that were not suitable for the other options in the above list.

#### Independent Variables

The body mass index (BMI) was calculated based on the pre-gestational weight provided by 246 pregnant women in the first ANC appointment in the study setting. Family income was assessed by asking the pregnant women about the monthly income of all family members. The amount was categorized into three categories based on the response of 285 pregnant women. The currency considered was the Brazilian minimum wage of 2019 (R$ 998,00), equivalent to approximately $241,00 (current currency).

### Sample Size

The sample size was calculated using a single prevalence of 14.0% (BiBintsene-Mpika et al., 2018). A sample error of 5% and a 95% confidence interval were considered. The analysis found that a sample of 186 pregnant women was able to represent the prevalence of self-medication during pregnancy. We did not consider the non-response rate.

### Statistical Analysis

A descriptive analysis was used to evaluate the categorical variables (frequency and percentage). The comparison between categorical variables and self-medication practice was analyzed using Fisher’s exact test and a Chi-square test. Quantitative variables (gestational age in weeks) were compared with self-medication practice with a Mann–Whitney *U* test. The possible variables associated with self-medication were assessed using univariate logistic regression analysis. Multivariate logistic regression analysis was performed considering the Stepwise criteria to assess where these variables were associated with self-medication. The significance level was set at 5% (*p* < 0.05). Statistical analyses were performed using SAS System for Windows (Statistical Analysis System, version 9.2, SAS Institute Inc., 2002–2008, Cary, NC, United States).

### Ethics

All women signed the informed consent before being included in the study. This research was approved by the Research Ethics Committee of the Faculty of Medical Sciences, UNICAMP (CAAE: 04012118.7.0000.5404). All procedure was carried out according to the Helsinki Declaration.

## Results

### Sociodemographic and Gestational Characteristics

Among the 297 pregnant women interviewed, 107 (36.0%) had practiced self-medication in the final 60 days before the first ANC appointment in a referral women’s hospital. The women were aged 18–52 years, with a mean of 30.9 ± 6.3 (±standard deviation (SD)). One hundred and sixty pregnant women (53.9%) were non-White, 209 (70.3%) had a partner, and 162 (54.5%) were employed (Table 1). Most pregnant women were in the second gestational trimester. The mean gestational age was 20.2 ± 7.7 weeks, and 220 (74.0%) pregnant women were multigravida. The most common diseases presented during pregnancy were hypertensive syndrome and any kind of diabetes, reported by 87 (29.2%) and 84 (28.2) pregnant women, respectively (Table 2).

**TABLE 1:** Sociodemographic, gestational characteristics and self-medication during pregnancy of 297 women.

Variables		Self-medication			
		Total, N = 297 (%)	Yes, n = 107 (%)	No, n = 190 (%)	*p* value
Age	<30	128 (43.1)	47 (43.9)	81 (42.6)	0.841
	30–39	143 (48.1)	52 (48.6)	91 (47.8)	
	≥40	26 (8.8)	8 (7.4)	18 (9.4)	
Skin color	White	137 (46.1)	51 (47.6)	86 (45.2)	0.690
	Non-White	160 (53.9)	56 (52.3)	104 (54.7)	
Marital status	With partner	209 (70.3)	77 (71.9)	132 (69.4)	0.652
	Without partner	88 (29.7)	30 (28.0)	58 (30.5)	
Occupation	Employed	162 (54.6)	63 (58.8)	99 (52.1)	0.456
	Housewives	111 (37.3)	35 (32.7)	76 (40.0)	
	Unemployed	24 (8.1)	9 (8.4)	15 (7.8)	
Education	Elementary school	49 (16.5)	11 (10.2)	38 (20.0)	**0.030**
	College/high school and university	248 (83.5)	96 (89.7)	152 (80.0)	
Family income^a^	0–2	208 (73.0)	75 (72.1)	133 (73.4)	0.481
	3–5	58 (20.3)	24 (23.0)	34 (18.7)	
	>5	19 (6.7)	5 (4.8)	14 (7.7)	
BMI*	Low weight	5 (2.0)	1 (1.1)	4 (2.4)	0.961
	Adequate	73 (29.7)	26 (30.5)	47 (29.1)	
	Overweight	75 (30.5)	26 (30.5)	49 (30.4)	
	Obesity	93 (37.8)	32 (37.6)	61 (37.8)	
Trimester	First trimester	64 (21.5)	35 (32.7)	29 (15.2)	**< 0.001**
	Second trimester	171 (57.6)	58 (54.2)	113 (59.4)	
	Third trimester	62 (20.8)	14 (13.0)	48 (25.2)	
Gravidity	1	77 (25.9)	20 (18.6)	57 (30.0)	**0.032**
	≥2	220 (74.1)	87 (81.3)	133 (69.8)	

a Family income was obtained using a sample of 285 women; BMI: body max index; BMI was calculated using a sample of 246 women; *p* value from Chi-square test and Fisher exact test. Significant *p* values are bold.

**TABLE 2:** Main health problems presented by pregnant women.

Health problem^a^	N (%)
Hypertensive syndrome during pregnancy	87 (29.2)
Diabetes mellitus during pregnancy	84 (28.2)
Thyroid disease	38 (12.7)
Collagen disease and antiphospholipid syndrome (APS)	25 (8.3)
Multiple pregnancy	22 (7.4)
Bad obstetric history (BOH) ^+^	20 (6.7)
Nervous system problems	19 (6.3)
Uterine anomaly and preterm labor	18 (6.0)
Infection during pregnancy	18 (6.0)
Pulmonary disease	14 (4.7)
Mental health	14 (4.7)
Obstetric complications	11 (3.7)
Diagnosis under investigation	18 (6.0)
Others	61 (20.5)

a More than one health problem per woman. +Examples of bad obstetric history found: perinatal deaths, malformations, and history of miscarriage. Others: non-specified health problems, 15 (5.0%); anemia, 7 (2.3%); non-specified cardiovascular and hematologic problems, 4 (1.6%); Crohn’s disease and bariatric surgery, 3 (1.0%) (each); history of orthopedic surgery, nephrolithiasis, history of renal lithiasis, hyperemesis gravidarum, history of cervical intraepithelial neoplasm, 2 (0.6%) (each); cirrhosis, chronic liver disease, problem in inguinal region, polycystic ovary syndrome, bilateral renal agenesis, intrauterine growth restriction, alcoholism, allergic rhinitis, kidney stone biliary lithiasis, cholestasis, keratoconus, chronic pain, fibromyalgia, history of hydatidiform spring, weight loss during pregnancy, history of splenectomy and colectomy, previous uterus neoplasm, non-specified surgery during pregnancy, 1 (0.3%) (each).

### Self-Medication Practice

One hundred and seven pregnant women (36.0%) had reported the practice of self-medication at least once in the final 60 days before their first ANC appointment in a referral women’s hospital. OTC drugs were the most used medication group in self-medication. Acetaminophen (Panda et al., 2016) (35.1%), metamizole (30 (28.0%)), and metamizole association (19 (17.7%)) were the most used medications in this group. Naphazoline (5 (4.6%)) and dimenhydrinate (4 (3.7%)) were the leading medications used in self-medication with POM, whereas guaco syrup (3 (2.8%)) was the most medication used in the MP group. Eleven (10.2%) pregnant women did not remember the name of the medications used in self-medication (Table 3).

**TABLE 3:** Main characteristics related to self-medication during pregnancy.

Medications used during self-medication^a^		N (%)
OTC drugs	Acetaminophen	38 (35.1)
	Metamizole	30 (28.0)
	Metamizole + association	19 (17.7)
	Ibuprofen	10 (9.3)
	Scopolamine	6 (5.6)
	Others^1^	21 (19.1)
POMs	Naphazoline	5 (4.6)
	Dimenhydrinate	4 (3.7)
	Omeprazole	3 (2.8)
	Others^2^	14 (14.4)
MP, vitamins, and phytotherapy	Guaco syrup	3 (2.8)
	Multivitamin	2 (1.8)
	Passionflower	2 (1.8)
	Others^3^	8 (7.2)
Do not remember the name of medication used		11 (10.2)
Indication for self-medication practice	Headache	63 (58.8)
	Nausea and vomiting	10 (9.3)
	Stomachache	8 (7.4)
	Flu	6 (5.6)
	Others^4^	73 (67.3)
Motivational factors	Considered a simple health problem	67 (62.6)
	Practicality	54 (50.4)
	Lack of access to health system	26 (23.4)
	Others^5^	13 (12.1)
Source or person who indicates self-medication practice	Own account (myself)	81 (89.0)
	Neighbor or friend	12 (11.2)
	Leftover from a previous treatment	11 (10.2)
	Family member	9 (8.4)
	Pharmacy	9 (8.4)
	Internet	6 (5.6)
	Others^6^	5 (4.5)

a More than one medication per woman. MP: medicinal plants. Others^1^: simethicone, 4 (3.7%); acetaminophen + association, 3 (2.8%); aluminum hydroxide + magnesium + simethicone, sodium bicarbonate + sodium carbonate + citric acid and bicarbonate, 2 (1.8%) (each); loratadine, dexchlorpheniramine, guaifenesin, diclofenac, sorbitol + sodium lauryl sulfate, magnesium carbonate, mineral oil, and *Saccharomyces cerevisiae*, 1 (0.9%) (each). Others^2^: meclizine, ondansetron, propranolol, 2 (1.8%) (each); Pantoprazole, metformin, tramadol, bromopride, amoxicillin, dexamethasone, metoclopramide, tranexamic acid, zolpidem, and cyclobenzaprine, 1 (0.9%) (each). Others^3^: *Peumus boldus* tea, 2 (1.8%); herbis tea, vitamin c, avocado leaf, Greek hay oil (aromatherapy), arnica gel, and clove tea, 1 (0.9%) (each). Others^4^: nasal congestion, colic, and general pain, 5 (4.6%) (each). Backache, heartburn, insomnia, toothache, and, cough 4 (3.7) (each); migraine, indigestion, allergy, and intestinal gas, 3 (2.8%) (each); myalgia, control diabetes mellitus, abdominal pain, intestinal constipation, urinary tract infection (UTI) and high blood pressure, 2 (1.8%) (each); treatment for polycystic ovary, kidney stone, leg pain, flaccidity, arrhythmia, tendonitis, hair loss, sinusitis, to menstruate, anxiety, diarrhea, low fetal weight, fever, and sore throat, 1 (0.9%) (each). Others^5^: previous experience with the medication, 5 (4.6%); discomfort caused by symptoms, 2 (1.8%); work, habit, curiosity, not getting the prescription, 1 (0.8%) (each). Others^6^: prescribed for another symptom, 2 (1.8%); habit, 2 (1.8%); and prescribed to a family member, 1 (0.9%). Those were cases where women reported more than one symptom, motivational factor, and indication for self-medication practice.

The most common indications for self-medication practice were headache (63 (58.8%)), nausea and vomiting (10 (9.3%)), and stomachache (8 (7.4%)). A simple health problem (67 (62.6%)) and practicality (64 (60.4%)) were motivational factors frequently reported by pregnant women who practiced self-medication, and the leading source for self-medication was “own account” (81 (89.0%)) followed by a neighbor or friend (12 (11.2%)) (Table 3).

Self-medication was associated with higher education (*p* = 0.033), first gestational trimester (*p* = <0.001), lower gestational age (*p =* <0.001), and gravidity (*p* = 0.030). A univariate logistic regression analysis was performed to assess the variables related to self-medication practice and higher education; gestational trimester and gravidity were determinant factors for self-medication (Table 4). Pregnant women with high-school (73 (68.2%) (OR = 2.10; 95% CI 1.01–4.37; *p* = 0.047)) or university-level (23 (21.5%) (OR = 2.48; 95% CI 1.05–5.86; *p* = 0.038)) education were more likely to practice self-medication than pregnant women with elementary school. Women in the first gestational trimester 35 (32.7%) (OR = 4.14; 95% CI 1.91–8.96; *p* = <0.001) and women with two or more pregnancies 87 (81.3%) (OR = 1.86; 95% CI 1.05–3.32; *p* = 0.034) were more likely to practice self-medication than pregnant women in the second and third gestational trimester and women in the first pregnancy, respectively.

**TABLE 4:** Univariate and multivariate logistic regression analysis of variables associated with self-medication during pregnancy (*n* = 297).

Variables	Self-medication		COR (95%CI)	*p* value	AOR (95%CI)	*p* value
	Yes	No				
*Age*						
<30	47	81	1.00	1	-	-
30–39	52	91	0.99 (0.60–1.62)	0.952	-	-
≥ 40	8	18	0.77 (0.31–1.90)	0.565	-	-
*Skin color*						
White	51	86	1.00	1	-	-
Non-White	56	104	0.91 (0.57–1.46)	0.690	-	-
*Marital status*						
With partner	77	132	1.00	1	-	-
Without partner	30	58	0.89 (0.53–1.50)	0.652	-	-
*Occupation*						
Housewives	35	76	1.00	1	-	-
Employed	63	99	1.38 (0.83–2.30)	0.214	-	-
Unemployed	9	15	1.30 (0.52–3.26	0.572	-	-
*Education*						
Elementary school	11	38	1.00	1	1.00	1
Secondary school	73	120	2.10 (1.01–4.37)	**0.047**	2.52 (1.17–5.43)	**0.018**
Superior level	23	32	2.48 (1.05–5.86)	**0.038**	2.82 (1.15–6.94)	**0.024**
*Family income* ^+^						
0–2	75	133	1.00	1	-	-
3–5	24	34	1.25 (0.69–2.27)	0.459	**-**	**-**
> 5	5	14	0.63 (0.22–1.83)	0.399	**-**	**-**
BMI*						
Low weight	1	4	1.00	1	-	-
Adequate	26	47	0.45 (0.05–4.26)	0.488	**-**	**-**
Overweight	26	49	0.96 (0.49–1.88)	0.904	**-**	**-**
Obesity	32	61	0.95 (0.50–1.80)	0.871	-	-
*Trimester*						
First trimester	35	29	4.14 (1.91–8.96)	**0.002**	3.61 (1.64–7.96)	**-**
Second trimester	58	113	1.76 (0.90–3.45)	0.242	1.51 (0.76–3.01)	**-**
Third trimester	14	48	1.00	1	1.00	-
*Gravidity*						
1	20	57	1.00	1	1.00	1
≥ 2	87	133	1.86 (1.05–3.32	**0.034**	1.96 (1.07–3.60)	**0.029**

COR: crude odds ratio; AAR: adjusted odds ratio; CI: confidence interval. Significant *p* values (*p* < 0.005) are bold.

The multivariate logistic regression analysis was performed considering education, gestational trimester, and gravidity and confirmed that these variables were significant factors associated with self-medication. Pregnant women with high-school (73 (68.2%) (OR = 2.52; 95% CI 1.17–5.43; *p* 0.018) or university-level (23 (21.5%) (OR = 2.82; 95% CI 1.15–6.94; *p* = 0.024)) education were more likely to practice self-medication when compared to women who had only attained primary school education. Pregnant women in the first gestational trimester (35 (32.7%) (OR = 3.61; 95% CI 1.64–7.96; *p* = 0.002)) and women with two or more pregnancies (87 (81.2%) (OR = 1.96; 95% CI 1.07–3.60; *p* = 0.029)) were more likely to practice self-medication than pregnant women in the second or third gestational trimester and in the first pregnancy, respectively.

## Discussion

Our study found a self-medication prevalence of 104 (36.0%). This finding is similar to the research performed in Ethiopia (Abeje et al., 2015), Nigeria (Adanikin and Awoleke, 2017), Iran (Afshary et al., 2015; Botyar et al., 2018), and Pakistan (Bohio et al., 2016), but higher than studies published in Peru (Miní et al., 2012), Serbia (Odalovic et al., 2012), Netherlands (Verstappen et al., 2013), and China (Liao et al., 2015). This difference could be due to the study setting, health policies regarding medication use, and the methods applied, such as the studied population and recording period considered to assess self-medication practice.

OTC drugs were the most used medication class in self-medication. This result may have been due to the relatively easy access to these medicines in Brazil and other countries. Acetaminophen was the most used medication in the OTC drugs group, and this finding is similar to previous studies. (Yusuff and Omarusehe, 2011; Odalovic et al., 2012; Cabut et al., 2017; Jambo et al., 2018; Zewdie et al., 2018). Beyond the possible association between prenatal exposure to acetaminophen and behavior problems in childhood (Liew et al., 2014; Liew et al., 2016; Stergiakouli et al., 2016; Bauer et al., 2018), acetaminophen can cause acute liver failure in mothers (Casey et al., 2020), leading to serious health problems, such as organ transplantation (Gill et al., 2002; Thornton and Minns, 2012), representing another risk to the fetus.

The use of MP and phytotherapy was reported at a lower frequency when compared to POMs and OTC drugs. According to the literature, the use of these products is common in rural areas, and women who are more highly educated are less likely to use this treatment (Bello et al., 2011). This finding provides an idea about why MP and phytotherapy were less used in our study; the Women’s Health Clinic is located in an urban area, and self-medication in our sample was common among women with a higher education level.

Although MPs were used less frequently by our sample, a study in Northeastern Brazil recorded a high prevalence regarding the use of this form of therapy. *Peumus boldus*, *Melissa officinalis*, *Matricaria chamomilla*, and *Mentha piperita L*. have abortifacient and teratogenic properties and were reported by Araújo et al*.* as frequently used MPs by pregnant women in Brazil (Araújo et al., 2016). It is important to highlight that teratogenic effects are related to different variables, such as gestational age at the exposure time and dose response, and MP use during pregnancy needs to be followed by a health care professional.

Headache was the most frequent indication for self-medication practice and this observation is similar to other studies (Bohio et al., 2016; Cabut et al., 2017; Alonso-Castro et al., 2018). Beyond hormonal changes and considering the high prevalence of diseases presented by our sample, the presence of headaches can be related to secondary causes, such as hypertension and pre-eclampsia. In this case, self-medication can contribute to a misdiagnosis (Bennadi, 2013), representing another risk beyond teratogenic effects, and pregnant women must be advised regarding these symptoms.

In our sample, the leading cause of self-medication practice was practicality, and a lack of health-system access was responsible for a minor self-medication prevalence. This finding reinforces that access to ANC services is not enough to prevent self-medication (Araújo et al., 2013), and this practice can occur even with health-system access. When asked about the indication related to self-medication practice, the leading answer was “myself.” This response could be related to the variables found to be associated with self-medication practice in our study, such as the number of previous pregnancies and education level.

Pregnant women with high-school or university-level education were more likely to practice self-medication when compared to pregnant women with elementary-school education. Although this finding is comparable with other studies (Afshary et al., 2015; Alonso-Castro et al., 2018), self-medication is generally related to lower levels of education (Bello et al., 2011; Garofalo et al., 2015; Marwa et al., 2018; Zewdie et al., 2018). This difference could be due to the study setting. Because Brazil is a developing country, people may have more access to drugs, and pregnant women with a higher level of education may feel more able to choose their own medicines.

Pregnant women in the first gestational trimester were more likely to practice self-medication than women in the second and third gestational trimester. During the first gestational trimester, women typically experience more symptoms and discomfort and tend to feel the need to use medicines more often; however, this is the more critical trimester regarding fetus damage related to medicine use (van Gelder et al., 2010).

Despite the methodological difficulty in establishing a relationship, some medications used in self-medication by our sample seem to have the potential to expose the fetus to teratogenic effects. As an example, some studies have demonstrated a possible teratogenic effect regarding ondansetron prenatal exposure and a risk of birth defects, such as cleft palate and cardiac defects, and its use is recommended by some authors as the last treatment option (Danielsson et al., 2014; Carstairs, 2016; Lavecchia et al., 2018). One study regarding self-medication among pregnant women found a significant relationship between self-medication during pregnancy and an increase in the US Food and Drug Administration (FDA) risk category (Adanikin and Awoleke, 2017). This finding highlights the need for close monitoring during ANC, mainly in the first gestational trimester, with appropriate instructions to minimize fetus exposure to teratogenic effects. The number of previous pregnancies was associated with self-medication. Women with two or more pregnancies were more likely to practice self-medication than women in the first pregnancy. These data are similar to the literature (Abeje et al., 2015; Beyene and Beza, 2018) and although there is a requirement for further studies, the experience of previous pregnancies seems to influence the women’s behavior in their subsequent pregnancies, particularly regarding medication use. This information can be useful to create prevention strategies regarding self-medication during the gestational period, mainly after the women’s first pregnancy.

The women’s knowledge about the risks of OTC drugs to the fetus was related to a slightly increased likelihood to practice self-medication during pregnancy (Beyene and Beza, 2018; Atmadani et al., 2020). There is a need to improve women’s knowledge regarding this topic to prevent self-medication during pregnancy. Based on our findings, this prevention strategy could be initiated during the women’s first pregnancy, preventing self-medication practice in future pregnancies.

Another alternative to minimize medication exposure during pregnancy, even through self-medication or prescription use, is to improve non-pharmacological treatment during this period. Regular exercise, fiber ingestion, and leg elevation are examples of non-pharmacological approaches recommended by WHO for the management of back pain, constipation, and edema, respectively (WHO, (2016)). Such alternative approaches should be encouraged and followed by health care professionals based on each women’s needs and medical history.

Beyond the presented alternatives to prevent self-medication and promote the rational use of drugs, pharmacists play an important role in improving medication safety during pregnancy, and these professionals should be aware of this topic so that they can more effectively guide pregnant women (Samuel and Einarson, 2011). As a suggestion for further studies, there is a need to evaluate women’s behavior before, during, and after pregnancy, mainly regarding medication use to improve prevention strategies for self-medication during this period. Because this study excluded pregnant women who were not fluent in Portuguese, there is a need to carry out research with this specific population, considering that difficulty in communication can impact health-system access, leading to self-medication practice.

Although the prevalence of self-medication in our survey was comparable to the literature, this study has some limitations. When practiced to treat a minor illness, self-medication can be rapidly forgotten, and the difficulty in remembering increases with long periods (Van den Brandt et al., 1991). Although the 60-day period considered to assess self-medication practice minimized recall bias, some pregnant women did not remember the name of the medications used during their self-medication practice, and the period considered excluded medications used by pregnant women in early periods. Because this study was performed in a public teaching hospital, it would be interesting for further research to assess self-medication practice in other services to examine the correlation between sociodemographic factors and self-medication practice. As for strengths, this study assessed self-medication practice among pregnant women of different ages, gestational trimesters, and comorbidities and included varying sociodemographic profiles. Beyond contributing to future research in the field, the factors associated with self-medication practice could help in the development of prevention strategies related to self-medication during pregnancy.

## Conclusion

The pregnant women were found to have practiced self-medication in the final 60 days before this survey, with the majority being OTC. Pregnant women with a higher education level, with two or more pregnancies, and in the first trimester were more likely to practice self-medication during pregnancy. Practicality was the main cause of self-medication, indicating that patient counseling is as important as ANC system access. There is a need to improve women’s knowledge regarding medication use during pregnancy.

## Data Availability

The datasets presented in this article are not readily available because of ethical considerations. Requests to access the datasets should be directed to GP, gabrielaper8@gmail.com.

## Abbreviations

WHO: World Health Organization; ANC: antenatal care; OTC: over-the-counter; POM: prescription-only medicine; MP: medicinal plant; STROBE: Strengthening the Reporting of Observational Studies in Epidemiology; SD: standard deviation; OR: odds ratio; IC: confidence interval; DRP: drug-related problem; ADR: adverse drug reaction.
