# Corrigendum: Self-Medication Among Pregnant Women: Prevalence and Associated Factors

**DOI:** 10.3389/fphar.2021.810762

**Published:** 2021-12-03

**Authors:** Gabriela Pereira, Fernanda Garanhani Surita, Amanda Canato Ferracini, Cinthia De Souza Madeira, Letícia Silva Oliveira, Priscila Gava Mazzola

**Affiliations:** ^1^ Faculty of Pharmaceutical Sciences, University of Campinas, Campinas, Brazil; ^2^ School of Medical Sciences, Department of Obstetrics and Gynecology, University of Campinas, Campinas, Brazil; ^3^ Graduate Program in Medical Sciences, School of Medical Sciences, University of Campinas, Campinas, Brazil; ^4^ Graduate Program in Gerontology, School of Medical Sciences, University of Campinas, Campinas, Brazil

**Keywords:** self-medication, pregnancy, women’s health, medication use, antenatal care

In the original article, we neglected to include the “Funder FAPESP, 2018/07707-6”. The new Funding statement reads as follows:

This study was financed in part by the Coordenação de Aperfeiçoamento de Pessoal de Nível Superior—Brasil (CAPES)—Finance Code 001 (GP), São Paulo Research Foundation (FAPESP) grant 2016/22335-2,2018/00070-2 (ACF), FAPESP grant 2018/07707-6 (PGM) and Brazilian National Research Council (CNPq) grant 301436/2017-7.

The authors apologize for this error and state that this does not change the scientific conclusions of the article in any way. The original article has been updated.

